# Solid immersion microscopy images cells under cryogenic conditions with 12 nm resolution

**DOI:** 10.1038/s42003-019-0317-6

**Published:** 2019-02-21

**Authors:** Lin Wang, Benji Bateman, Laura C. Zanetti-Domingues, Amy N. Moores, Sam Astbury, Christopher Spindloe, Michele C. Darrow, Maria Romano, Sarah R. Needham, Konstantinos Beis, Daniel J. Rolfe, David T. Clarke, Marisa L. Martin-Fernandez

**Affiliations:** 1 Central Laser Facility, Research Complex at Harwell, Science and Technology Facilities Council, Rutherford Appleton Laboratory, Harwell, Didcot, Oxford, OX11 0QX UK; 2 Diamond Light Source, Harwell Science and Innovation Campus, Didcot, OX11 0DE UK; 3 Department of Life Sciences, Imperial College London, London, SW7 2AZ UK; 4 Research Complex at Harwell, Rutherford Appleton Laboratory, Didcot, OX11 0FA UK

## Abstract

Super-resolution fluorescence microscopy plays a crucial role in our understanding of cell structure and function by reporting cellular ultrastructure with 20–30 nm resolution. However, this resolution is insufficient to image macro-molecular machinery at work. A path to improve resolution is to image under cryogenic conditions. This substantially increases the brightness of most fluorophores and preserves native ultrastructure much better than chemical fixation. Cryogenic conditions are, however, underutilised because of the lack of compatible high numerical aperture objectives. Here, using a low-cost super-hemispherical solid immersion lens (*super*SIL) and a basic set-up we achieve 12 nm resolution under cryogenic conditions, to our knowledge the best yet attained in cells using simple set-ups and/or commercial systems. By also allowing multicolour imaging, and by paving the way to total-internal-reflection fluorescence imaging of mammalian cells under cryogenic conditions, s*uper*SIL microscopy opens a straightforward route to achieve unmatched resolution on bacterial and mammalian cell samples.

## Abstract (web summary)

Lin Wang et al. present a new super-resolution modality using a super-hemispherical immersion lens. They achieve a 12 nm spatial resolution in cells under cryogenic conditions, which offers the technical means to study bacterial and mammalian cell samples at molecule localisation length-scales.

## Introduction

To fully understand the cellular function, we must combine cellular ultrastructure information with knowledge of protein interaction networks at the molecular level. Super-resolution fluorescence microscopy has underpinned our understanding of interacting molecular networks in cells. Techniques such as structured illumination microscopy (SIM)^1^, stimulated emission depletion (STED) microscopy^2^, and single molecule localization microscopy (SMLM) (including stochastic optical reconstruction microscopy (STORM)^3^, photoactivated localization microscopy (PALM)^4^, and fluorescence photoactivated localization microscopy (FPALM)^5^) image cellular processes at resolutions in the 20–100 nm range. However, when the goal is to ascertain structure–function relationships, the challenge is to improve resolution by at least ~2-fold, making it comparable with the typical size of interacting macro-molecular units (~10 nm).

Theoretically, resolution can be improved substantially by exploiting SMLM techniques under cryogenic conditions. In SMLM, resolution depends on the precision with which individual molecules can be localized^6,7^. This depends on the number of photons emitted by the sample, which substantially increases under cryogenic conditions, the number of photons collected by the objective lens, and its numerical aperture (NA)^6,7^. NAs >1 and preferably >1.4 are required to achieve high resolution, but these need immersion fluids to couple the sample to the objective. Liquid media freeze at cryogenic temperatures, so dry objectives (NA ≤ 0.9) are mostly used in this case, with the disadvantage of lower resolution. If this could be overcome, the use of cryo-fixation could become routine in super-resolution microscopy, with the added benefit that rapid freezing is more effective than chemical fixation at preserving ultrastructure and minimizing artefacts, as demonstrated by electron microscopy (EM)^8^.

There are several examples in the literature of super-resolution under cryogenic conditions. The increased photon yield under cryogenic conditions allowed Kaufmann et al.^9^ to compensate for the low NA of the objective to achieve sub-diffraction limited resolution (~125 nm) on green fluorescence protein (GFP)-labelled samples. Liu et al.^10^ attained ~46 nm resolution using DRONPA, a protein 2.5 times brighter than GFP. Other set-ups relied on custom-built stages to incorporate cryofluids (immersion fluids that remain liquid at cryogenic temperatures); Nahmani et al.^11^ achieved ~35 nm, close to the resolution currently possible at room temperature in cell samples with simple and/or commercial set ups, by using a water-immersion objective and a methanol/propanol mixture as the immersion fluid (NA = ~1.2). A similar approach was taken by Faoro et al.^12^, but this set-up was not applied to super-resolution microscopy. Highly specialized cryo-STORM systems functioning at liquid helium temperature have yielded ~Angstrom localization precision in isolated molecules^13,14^. However, these complex, custom-built cryo-stages have not yet been employed in cell imaging.

We have solved this challenge using super-hemispherical solid immersion lenses (*super*SILs), truncated balls made of solid materials of high refractive index that fill the gap between the objective and the sample, eliminating the requirement for coupling fluids^15–17^. By using a *super*SIL to couple the sample to a dry objective, the effective NA of the latter is enhanced up to the value of the refractive index of the *super*SIL. We and others previously demonstrated the enhanced resolution of *super*SIL microscopy at room temperature^18–22^. The breakthrough here was the realization that the high NA delivered by *super*SILs is eminently compatible with cryogenic conditions and particularly suited to STORM techniques, for which, to the best of our knowledge, they have not been used before despite the critical dependence of STORM on NA.

We describe a STORM set-up that uses solid immersion technology and cryogenic conditions to achieve 12 nm resolution on bacterial cells. Importantly, this was achieved using a low-tech set-up that can be implemented by any laboratory. By achieving NAs >1.45, we also remove the barrier to total internal reflection fluorescence (TIRF) imaging of mammalian cells under cryogenic conditions. Because our set-up outperformed in some respects a much more expensive state-of-the-art STORM system at room temperature, we conclude that *super*SIL microscopy could become the method of choice for straightforward exploitation of nanoscale cell imaging in microscopy at any temperature.

## Results

### A cryo-compatible *super*SIL-based STORM microscope

**Fig. 1:**
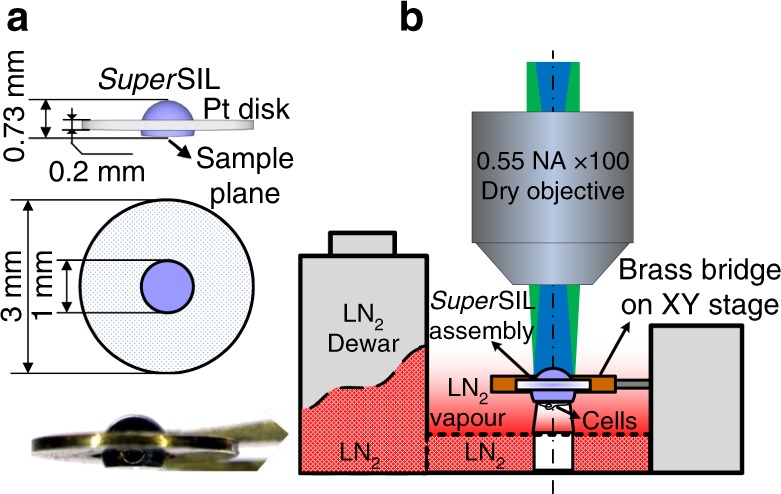
Schematic of superSIL microscope. **a** Side-view (top of the panel) and top-view (centre of the panel) of *super*SIL assembly. The side view shows the location of the sample plane (aplanatic surface of the *super*SIL). The photo of an assembly is at the bottom of the panel. **b** Schematic of key components in the microscope. An upright microscope configuration was employed. The blue shading illustrates the Köhler illumination laser beam and the green shading indicates the fluorescence emission. (For more info, see Supplementary Figs. 1–3)

The key components are shown in Fig. 1. The optics employed to deliver and collect light consist of a 0.55 NA dry objective (Mitutoyo 100× Plan Apo SL Infinity Corrected) and a 1 mm diameter cubic zirconia *super*SIL (effective NA = 2.17). An achromatic doublet lens (200 mm focal length) was used as a tube lens before the EMCCD detector camera (Andor, iXon+ DU-897) (Supplementary Fig. 1). The *super*SIL was mounted into the central hole of a platinum disk using a thermally conductive cryo-adhesive (Loctite Stycast 2850 FT) (Supplementary Fig. 2). *Super*SIL assemblies are robust and inexpensive (~US$20), and can be reused by cleaning the surfaces.

The *super*SIL assembly is compatible with the standard EM grid-holder of an off-the-shelf liquid nitrogen (LN_2_)-cooled cryo-stage (Linkam, CMS-196). Samples were adhered to the flat surface of the *super*SILs and plunge-frozen together in liquid ethane (Supplementary Fig. 3). The convex surfaces of the *super*SILs faced upwards to the objective, and the flat surfaces faced downwards (Fig. 1b). *Super*SIL assemblies thus play at cryogenic conditions the role played by liquid immersion media at room temperature. The assemblies were mounted on top of the brass bridge in the cryo-stage and translated in XY directions for fine positioning. The cryo-stage is self-contained with a built-in LN_2_ reservoir, and can be easily integrated into any conventional upright fluorescence microscope. Frozen samples were kept at 77 K by the LN_2_ vapour surrounding the brass bridge of the cryo-stage.

### *Super*SILs can increase the resolution of STORM

The best theoretical localization precision value (*σ*), and therefore resolution, achievable in STORM imaging is effectively determined by

1 $$\sigma = \frac{S}{{\sqrt N }}$$

where $S \approx \frac{{0.75\lambda }}{{\pi NA}}$ is the standard deviation of a Gaussian function approximating the point spread function (PSF) of the microscope, *N* is the number of photons, and *λ* the imaging wavelength^6,7^. We speculated that the intrinsic properties of *super*SIL optics, namely their light collection efficiency^23,24^ and high NA, together with the increased photon budget at cryogenic conditions^10^, could deliver unprecedented resolution without requiring custom-made cryo-stages or other cumbersome, highly-specialized systems.

**Fig. 2:**
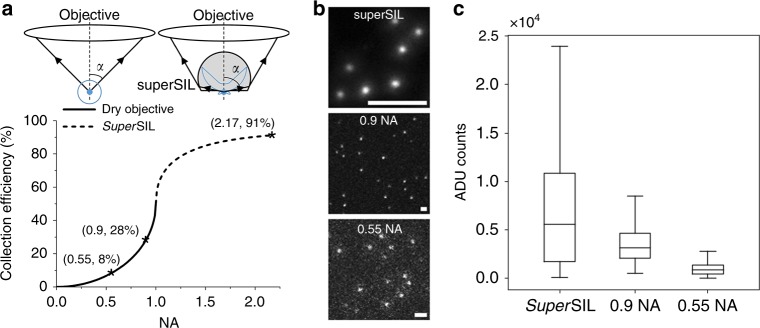
Comparison of collection efficiency in *super*SIL and standard objectives. **a** Models and simulations of collection efficiency in conventional fluorescence microscopy using dry objective lenses and *super*SIL microscopy. Schematics of ray propagation from a single fluorophore (blue dots) in conventional (top left) and *super*SIL microscopy (top right). The fluorescence emission distribution is depicted by the blue curves. Collection efficiency versus NAs from simulations is plotted in the graph at the bottom, in which the solid and dashed curves illustrate conventional and *super*SIL microscopy cases, respectively. **b** Representative images of 100 nm diameter fluorescent beads taken using the *super*SIL, 50 × 0.9 NA objective and 100 × 0.55 NA objective on the same microscope platform with identical filters, camera settings and laser illumination power. Scale bars: 2 µm. **c** The box chart showing the distribution of intensities from multiple beads fitted with Gaussian profiles, corrected for changes in laser power density arising from the different magnifications. The box extends from the lower to upper quartile values of the data, with a black line at the median. The median of intensity was 5573, 3162 and 862 in the case of *super*SIL, 0.9 NA and 0.55 NA objective, respectively. The whiskers extend from the box to show the range of data falling within the 1.5× inter-quartile range from the quartiles

The reason for the enhanced light collection efficiency of *super*SILs is the electromagnetic coupling to optically dense material via evanescent fields, described in detail by Yoshita et al.^24^. To illustrate this, we carried out simulations using the known fraction of light collected in a microscope from an isotropic light source^25^:

2 $$\frac{{\mathrm{\Omega }}}{{4\pi }} = \frac{1}{2}\left[ {1 - \sqrt {1 - \left( {NA/n} \right)^2} } \right] = \frac{1}{2}\left( {1 - {\mathrm{cos}}\,\alpha } \right)$$

where *NA* is the numerical aperture of an objective lens, and *n* is the refractive index of immersion medium. Fundamentally, the collection efficiency is determined by *α* which is half the maximal light collection angle (Fig. 2a).

In optical microscopes using dry objective lenses, collection efficiency increases rapidly with higher NAs (Fig. 2a, black solid curve), with a theoretical maximum value of 50% at *α* *=* 90°, i.e. 2*π* solid angle, in the case of an isotropic fluorescence dipole emitter (Fig. 2a, blue curves in the conventional fluorescence microscopy illustration). For example, the theoretical collection efficiency is 8% and 28% with NAs of 0.55 and 0.9, respectively. In practice, the maximum collection efficiency of dry objectives is limited to ~30% because severe aberrations arise from off-axis rays with larger NAs^26^. When coupled with a *super*SIL, a dry objective lens (i.e. NA ≥ 1/*n*, where *n* is the refractive index of the *super*SIL material) can collect light propagating with a maximum 90° polar angle^23,24^. This means that collection efficiency is effectively determined by the directional fluorescence emission pattern of a dipole emitter at a dielectric surface (Fig. 2a, blue curves in the *super*SIL microscopy illustration). A fluorophore in close proximity to a dielectric surface emits many more photons towards the dielectric medium due to stronger electromagnetic coupling to the medium with higher refractive index via an evanescent field^27^. Considering a fluorophore is in direct contact with the flat surface of a *super*SIL and there is no reflection loss at the surfaces, we simulated the collection efficiency versus effective NAs, determined by the refractive indices of the *super*SIL materials (Fig. 2a, black dashed curve). The theoretical collection efficiency in the *super*SIL microscope is 91%, which is 11.4-fold and 3.3-fold larger than those in the microscope using 0.55 NA and 0.9 NA dry objectives.

The theoretical predictions of collection efficiency from the three NAs were tested experimentally using the set up in Fig. 1 under otherwise identical conditions. The results show that the *super*SIL collected 6.5-fold and 1.8-fold more photons compared to 0.55 NA and 0.9 NA dry objectives (Fig. 2b, c). This is in agreement within errors with the predicted values (Fig. 2a). The discrepancy comes from three sources: (1) Reflection loss from *super*SIL surfaces; (2) The smaller portion of fluorescence emission directed into the *super*SIL as the samples, i.e. 100 nm beads, were effectively 50 nm away from the surface. (3) More fluorescence emission collected in the conventional microscope measurement because the coverslips introduced directional emission at the glass–air interface. Nevertheless, we validate in our set-up the previously predicted higher photon collection efficiency of *super*SIL. This means that, according to Eq. (1), the enhancement in photons collected by the 2.17 NA *super*SIL would increase the achievable resolution by ~$\sqrt N = 2.5 - {\mathrm{fold}}$ and ~$\sqrt N = 1.3 - {\mathrm{fold}}$ compared to objectives with NAs of 0.55 and 0.9, respectively.

STORM resolution is also inversely proportional to the NA (Eq. (1)). In the case of a *super*SIL-dry objective combination, the effective NA is: $NA_{eff} = n^2NA$, subject to $NA_{max} = n.$ The maximum $NA_{eff}$ is equal to the refractive index of the *super*SIL employed (2.17 in this case). Given this, one would expect enhancements in resolution of 3.9-fold and 2.4-fold from the larger NA of the *super*SIL system compared to standard dry objectives with NAs = 0.55 and 0.9, respectively.

Combining the two sources of resolution enhancement, namely the larger NA and the increase in photon collection, given Eq. (1), one would expect the localization precision that can be obtained using the combination of *super*SIL and dry objective to be ~10-fold and ~3-fold better than that obtainable with objectives with NAs of 0.55 and 0.9, respectively.

### *Super*SIL images plunge-frozen bacteria at 12 nm resolution

**Fig. 3:**
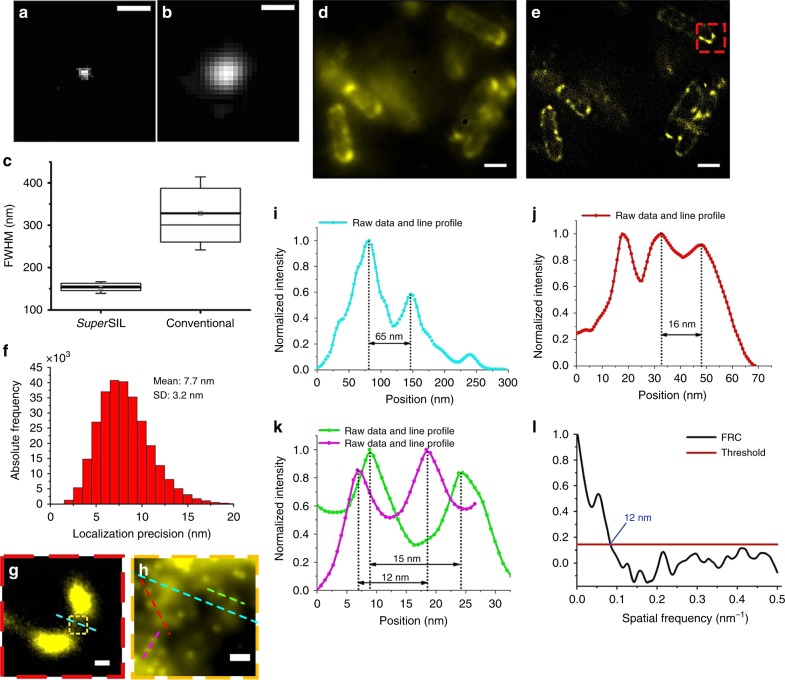
S*uper*SIL resolution characterization and imaging of *E. coli* cells under cryogenic conditions. Representative images of sub-diffraction limited objects from **a** the *super*SIL microscope and **b** the specialist system. Scale bar: 0.5 µm. **c** Box charts of FWHM of the PSFs for the *super*SIL and specialist systems showing the 25th and 75th percentiles of the data points. The thick line with a square at the center shows the mean value; the thin line in the box shows 50th percentile. The mean of FWHM was 153 ± 14 and 328 ± 86 nm in the case of *super*SIL and 0.9 NA objective, respectively. The whiskers show the standard deviation. **d** ATP-binding cassette (ABC) transporter protein PH1735 fused with EGFP in *E. coli* cells imaged in wide-field *super*SIL microscopy and **e** *super*SIL STORM. **f** Localization precision histogram from the image in **e**. **g** The enlarged image of the region of the cell indicated by the red dashed border box in **e**. **h** The enlarged image of the small region in the cell indicated by the orange dashed border box in **g**. **i** Line profile of the cross-section of two PH1735 protein clusters indicated by the cyan dashed lines in **h**. **j** Line profile of the cross-section of two PH1735 protein clusters closer to each other indicated by the red dashed line in **h**. **k** Line profiles of the cross-section of two adjacent single molecules (magenta and green) indicated by the magenta and green dashed lines in **h**. **l** FRC curve revealing the 12 nm resolution in the region shown in **h**. Scale bars: 1 μm (**d**, **e**), 100 nm (**g**) and 20 nm (**h**)

The first step to characterize the performance of the *super*SIL microscope was measuring the size of the PSF in the focal plane of object space. We acquired 500 photoluminescence images of the sparse cubic zirconia defect spots present on the flat surface of the *super*SILs (Supplementary Fig. 4). These intrinsic defects are single point emitters^28^, located at the aplanatic plane of the *super*SIL, which is the position with the fewest optical aberrations^18^. The full width at half maximum (FWHM) of the PSF of these images therefore gives an estimation of the best achievable wide-field, diffraction-limited, lateral resolution^29^. Representative images and statistical results show a PSF size of 153 ± 14 nm (Fig. 3a, c), in agreement with that previously reported^19^. The ~25% discrepancy between experiment and theory (118 nm predicted using the Houston criterion^30^ given an effective NA of 2.17) arises from the inefficiency of collecting rays propagating at angles approaching 90° from the optical axis^18^.

For comparison, we evaluated the PSF of a standard off-the-shelf cryo-compatible system, namely an FEI CorrSight fluorescence microscope with a standard dry objective (ZEISS EC Plan-Neofluar 40×/0.9 Pol M27), similar to that used by Kaufmann et al.^9^. A representative image and the statistical analysis of the PSF measurement using sub-diffraction limited 100 nm fluorescent beads (ThermoFisher, TetraSpeck) are shown in Fig. 3b, c. These results report a mean PSF size of 328 ± 86 nm, consistent with the theoretical value of 290 nm at the wavelength of 515 nm, according to Houston criterion^30^.

To explore the resolution improvement that could be achieved by combining *super*SIL microscopy and STORM we compared its performance with that of the CorrSight. We imaged in LN_2_ vapour, plunge-frozen *Escherichia coli* cells expressing the ATP-binding cassette (ABC) transporter PH1735, a putative multidrug transporter^31^, fused to enhanced GFP (EGFP), which displayed robust single molecule blinking behaviour at cryogenic temperature (Supplementary Fig. 5A, 5B). Representative wide-field and STORM images from the *super*SIL set up are shown in Fig. 3d, e. Images from the off-the-shelf system are shown in Supplementary Fig. 5C.

To reach quasi-equilibrium between fluorescent on and off states of the EGFP molecules, we delivered the same 1.1 kW cm^−2^ laser power density at 488 nm wavelength to the sample plane of both the *super*SIL and the off-the-shelf systems. Interestingly, the substantially larger photon delivery and collection efficiency of the *super*SILs allowed us to use a laser power of 5.55 mW, whilst for specialist cryo-STORM imaging we required a laser power of 60 mW. Thus, cryo-*super*SIL not only unlocks the potential to perform nanoscale STORM under cryogenic conditions, but enables the use of low-power, low-cost lasers.

Relevant to comparing their performance in STORM imaging, the *super*SIL and FEI CorrSight systems used different emission filters. The *super*SIL set-up employed a 512/25 nm bandpass filter, which transmits 57% of the fluorescence from EGFP (Supplementary Fig. 6A). The off-the-shelf system employed a 496 nm long pass filter that transmits 87% of the fluorescence (Supplementary Fig. 6B). Also relevant, the *super*SIL and FEI CorrSight systems used different detectors, an EMCCD in the *super*SIL microscope and a scientific CMOS (sCMOS) camera (Hamamatsu, ORCA-Flash4.0 V2) in the FEI CorrSight. We calibrated the detector settings used to image *E. coli* and found the EMCCD/sCMOS photon detection efficiency ratio was 1.2:1 (Supplementary Fig. 6E), showing that the EMCCD detector is 20% more efficient at the settings employed.

The ratio of localization precision between the *super*SIL and the FEI CorrSight settings predicted by Eq. (1) is (NA$\sqrt N$)/(NA′$\sqrt {N\prime }$). Substituting the 2.17 NA and 0.9 NA′ values of the two systems, the number of photons (*N* and *N*′) collected by the lenses, and considering the different filter transmissions and detector responses, the localization precision delivered by the *super*SIL set-up in the conditions described above should be ~2.8-fold better than that of the FEI CorrSight. The localization precision we obtained with the *super*SIL system is *σ*  = 7.7 ± 3.2 nm (Fig. 3f) and that obtained with the CorrSight is *σ*′ = 35.7 ± 9.4 nm (Supplementary Fig. 5C, D). The latter value is consistent with that previously achieved in comparable conditions^9^. These results are consistent within errors with theory predictions.

We note that in the case described above, the localization precision enhancement delivered by *super*SIL entirely depends on its larger NA. This is because, instead of the long pass filter used in combination with the 0.9 NA of the FEI CorrSight, a 25 nm bandpass filter was required to minimize the chromatic aberration of the *super*SIL (choices of emission filters were discussed and demonstrated in earlier work^18,19^). This means that the increase in collection efficiency of the *super*SIL with respect to the 0.9 NA objective (1.6-fold) is cancelled out by reduced filter transmission (0.65-fold). Therefore, in one colour imaging, when long pass filters can be used, one would expect a ~2.4-fold improvement of resolution using *super*SILs.

To illustrate the resolution of the cryo-*super*SIL system, in Fig. 3g we show an expanded view of the area within the red box in Fig. 3e. The orange box in Fig. 3g was further expanded in Fig. 3h, showing single PH1735-EGFP molecules embedded in the membrane together with larger features. The profiles of fluorescence intensity across the areas marked in Fig. 3h by dashed lines reveal two features separated by ~65 nm (Fig. 3i), two features separated by ~16 nm (Fig. 3j), and two pairs of molecules separated by ~12 and ~15 nm (Fig. 3k). This resolution is consistent with the observed localization precision of 7.7 nm. We also evaluated resolution using the Fourier Ring Correlation (FRC) method^32^. We used a fixed correlation threshold equal to 1/7 ≈ 0.143, the most appropriate for localization microscopy images, and identified a spatial frequency threshold valued at 0.084 nm^−1^ (Fig. 3l), revealing an FRC resolution of 12 nm. PH1735 is a homodimeric ABC transporter (thus, fused with two EGFP molecules), and these features probably belong to a full transporter. Taking into account the linker length between the PH1735 and EGFP, the measured separation of 12–15 nm probably represents a nucleotide-free inward-open transporter, which is consistent with distances measured on other ABC transporters such as McjD^33^. To our knowledge, the combination of the <8 nm localization precision and 12 nm resolution here demonstrated is the highest to date on intact cells. Our ability to resolve individual proteins in a dimer shows that the resolution of cryo-*super*SIL STORM is adequate to probe macro-molecular organization in cells.

**Fig. 4:**
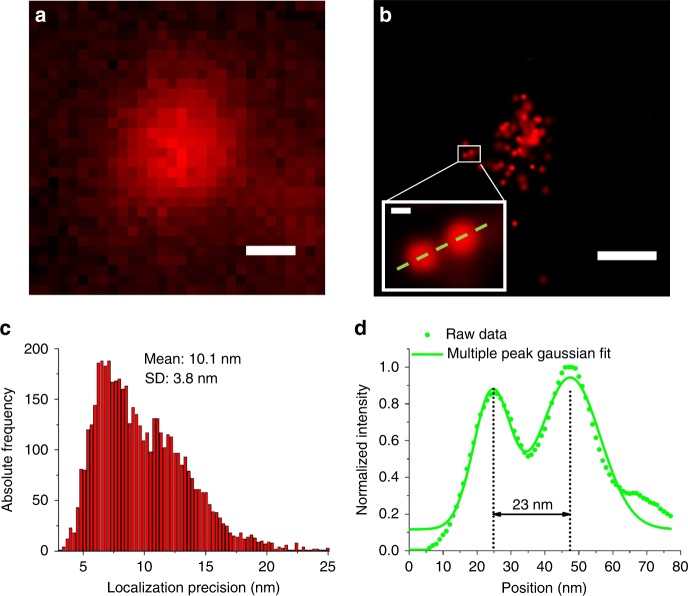
Resolution evaluation of cryo-*super*SIL STORM using DNA origami nanorulers. Images of ATTO 647N nanorulers from (**a**) wide-field cryo-*super*SIL fluorescence microscopy and **b** cryo-*super*SIL STORM (Scale bars: 200 nm). A nanoruler image is shown in the magnified inset within the white border box in **b**. Scale bar: 10 nm. **c** Localization precision histogram from the cryo-*super*SIL STORM image. **d** Line profile of the cross-section of a nanoruler (green dots), indicated by the green dashed line in the magnified inset in **b**, and its Gaussian fit (green line)

To objectively verify the resolution of the *super*SIL under cryogenic conditions, we imaged plunge-frozen ATTO 647N-based calibration samples of DNA origami nanorulers (GattaQuant)^34^, illuminated with 11.17 mW laser power (*λ* = 642 nm), resulting in a power density of 2.2 kW cm^−2^. Representative wide-field and STORM images are shown in Fig. 4a, b. Individual nanoruler structures can be observed in the cryo-*super*SIL STORM image, demonstrating a localization precision of 10.1 ± 3.8 nm (Fig. 4c). An example of the mark-to-mark distances from the nanorulers in the image of 23 nm magnitude is shown in Fig. 4d.

### Two-colour cryo-*super*SIL STORM imaging of bacterial cells

Two-colour imaging is required to study inter-molecular interactions. To investigate the possibility of two-colour imaging, we imaged plunge-frozen *E. coli* cells expressing the antibacterial peptide ABC transporter McjD fused with EGFP^33,35–37^, in which cell membranes were stained with the red fluorescent probe DiSC_3_(5). The DiSC_3_(5) dye is cationic and labels putative inner membrane nanodomains, i.e. the fluid lipid network^38^, which consists of negatively charged phospholipids.

**Fig. 5:**
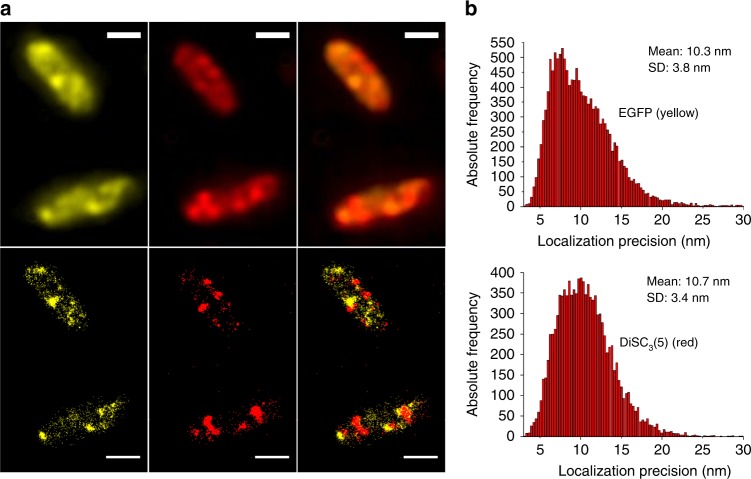
S*uper*SIL multi-colour imaging under cryogenic conditions. **a** McjD-EGFP (yellow) and membrane-DiSC_3_(5) (red) in *E. coli* cells imaged in (top) wide-field cryo-*super*SIL microscopy and (bottom) cryo-*super*SIL STORM. The overlays of two-colour images are shown in the right-side column in each row. Scale bar: 1 µm. **b** The histograms of localization precision of (top) EGFP and (bottom) DiSC_3_(5)

We used laser powers of 1.1 and 2.2 kW cm^−2^ in the 488 and 642 nm channels, respectively. Two-channel imaging was sequential, with the same camera setting (30 ms exposure time per frame, 10 MHz 14 bit EM amplifier readout rate, 5.2× preamp setting and 300 EM gain). Features detected in both channels in wide-field fluorescence images were used as markers to align two-colour super-resolution images during image post-processing. As under cryogenic conditions, 25 nm bandpass emission filters were used to minimize chromatic aberration (Supplementary Fig. 6A). We note, however, that, unlike in one colour imaging, where long pass filters are possible, in two colour imaging 25 nm bandpass filters are commonly used in conventional set-ups. Thus we would expect no significant losses in resolution from the requirement of 25 nm bandpass filters in *super*SIL set-ups, at least in the shorter wavelength channel. This is confirmed in our set-up which gave localization precisions of 10.3 ± 3.8 nm in the yellow channel and 10.7 ± 3.4 nm in the red channel (Fig. 5b). Images reveal the distribution of McjD proteins (*yellow*) and the organization of the fluid lipid network (*red*) (Fig. 5a), the latter displaying perceptible helical structures in agreement with previous findings^39^. A high degree of segregation is observed between the McjD and the lipid network.

In these proof-of-concept experiments, different colour images were collected sequentially with the same camera, as the longitudinal axial chromatic aberration required refocusing when changing imaging colours. We are currently implementing modifications to the microscope by splitting the emission path to acquire the two colour images simultaneously, and also deploying fiducial markers, which should achieve image correlation accuracies comparable to those previously shown^40^.

### Cryo-*super*SIL imaging of mammalian cells

Super-resolution microscopy under cryogenic conditions has been incompatible with TIRF illumination, because in cryogenic conditions the available objectives have NA ≤ 1.2, and to create the evanescent fields required by TIRF, NAs > 1.4 are required^41,42^. This has precluded the application of super-resolution microscopy to the investigation of cryo-frozen mammalian cells.

Similarly to liquid immersion objectives, the depth of focus of the combination of the *super*SIL and its partner dry objective lens is inversely proportional to the square of the effective NA^29^. This results in a narrow depth of focus, and thus similar properties to TIRF. We previously demonstrated TIRF imaging using *super*SILs but only at room temperature^19^. Under cryogenic conditions we found that the resolution of the *super*SIL changes by the approximately constant value of ±15% throughout the depth range of 10–100 nm (Supplementary Fig. 7). From this we conclude that the resolution of the *super*SIL-dry lens combination is maintained up to separations of 100 nm from the flat surface of the lens, a depth is comparable to that achievable by TIRF. This paves the way to investigate crucial processes in mammalian cells that require TIRF imaging under cryogenic conditions.

**Fig. 6:**
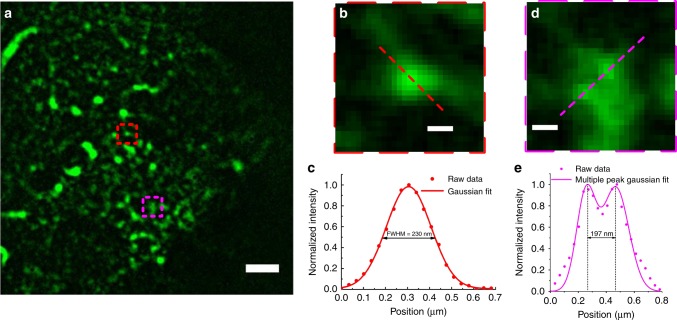
Wide-field superSIL microscopy under cryogenic conditions. **a** CHO cells expressing EGFR labelled with Alexa Fluor 488. **b** A cluster of EGFR indicated by the red dashed border box in **b**. **c** The profile of the cross-section of the EGFR cluster (red dots) indicated by the red dashed line in **b**, and its Gaussian fit (red line). **d** Two adjacent EGFR clusters indicated by the magenta dashed border box in **a**. **e** The profile of the cross-section of the two adjacent EGFR clusters (magenta dots) indicated by the magenta dashed line in **d**, and their Gaussian fits (magenta line). Scale bars: 2 μm (**a**) and 0.2 μm (**b**, **d**)

To verify in cells that the predicted resolution is maintained in the basal periplasmic section adjacent to the lens surface, we imaged plunge-frozen Chinese hamster ovary (CHO) cells expressing the epidermal growth factor receptor (EGFR), a key molecule in cancer research^43^. EGFR was labelled with Alexa Fluor 488 (Thermo Fisher). To extract areas of highest resolution from the wide-field images, we used a ‘rolling ball’ background reduction algorithm during image post-processing^44^. As shown in Fig. 6a, the TIRF-like appearance of the image is apparent^45^. A small EGFR cluster in the cryo-*super*SIL image showed an apparent width of 230 nm (Fig. 6b, c). Two adjacent clusters 197 nm apart are also clearly distinguishable (Fig. 6d, e). These values are indistinguishable to those predicted at depths from 10 to 100 nm (Supplementary Fig. 7), showing that conventional TIRF would be eminently possible at cryogenic temperatures using *super*SIL optics.

It is worth noting that the depth of focus of a *super*SIL microscope depends on the refractive index of the *super*SIL material, allowing a degree of depth ‘tuning’. *Super*SIL materials of lower refractive indexes (e.g. Quartz) could be used to increase the depth of focus approximately 2.2-fold (Supplementary Table 1). This is an important consideration to exploit *super*SIL imaging in correlative light and electron microscopy (CLEM), where cell lamella thickness is in the range of 50–300 nm^46^. A suitable technique for nanoscale resolution under cryogenic conditions, like cryo-*super*SIL STORM, can provide true complementarity between EM and fluorescence microscopy, crucial to realize the promise of CLEM in biology^47^.

### Ambient *super*SIL STORM outperforms off-the-shelf systems

**Fig. 7:**
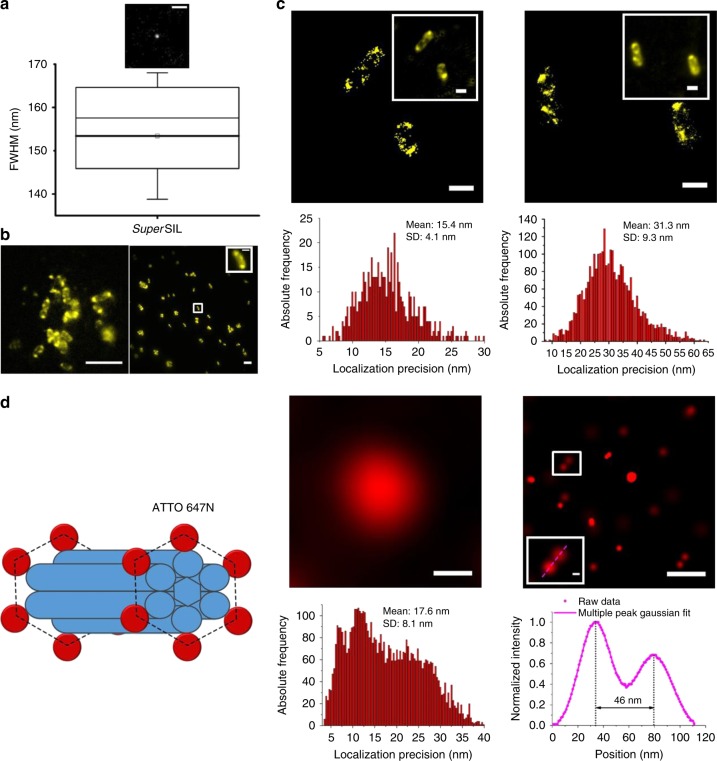
S*uper*SIL resolution performance at room temperature. **a** Image of a point object (top). Scale bar: 0.5 µm. The box chart (bottom) of the full width half maximum (FWHM) measurements of images from point emitters. The boxes show the 25th and 75th percentiles of the data points. The thick line with a square at the center shows the mean value; the thin line in the box shows 50th percentile. The mean of FWHM was 153 ± 15 nm. The whiskers show the standard deviation. **b** Comparison of wide-field images of live McjD-EGFP in *E. coli* cells. The *super*SIL image (left) has ×471  magnification, while the specialist microscope (right) has ×100 magnification. For comparison the inset contains a ×4.71 scaled-up image of a cell. Scale bar: 5 µm, and 1 µm in the inset. **c** STORM images (top) and localization precision histograms (bottom) of live McjD-EGFP *E. coli* cells in *super*SIL STORM (left) and specialist STORM (right). The insets show wide-field fluorescence images of the cells. Scale bar: 1 µm. **d** Resolution evaluation of STORM imaging in the specialist microscope. Left: Schematic of DNA origami nanorulers labelled with ATTO 647N dye molecules. Right: image of a field of nanorulers obtained from wide-field (top left) and STORM (top right). Scale bar: 200 nm. The STORM image of a nanoruler is shown in the inset, indicated by the white-border box. Scale bar: 20 nm. Bottom left: Localization precision histogram from the STORM image of the nanorulers. Bottom right: Profile of the cross-section of the nanoruler (magenta dots) in the magnified inset, and its Gaussian fit (magenta line)

To compare with the resolution improvement at cryogenic conditions, we characterized *super*SIL microscope performance at room temperature. We first verified that the freezing procedure had not altered the spectral dispersion and thermal expansion properties of the CZ *super*SILs (Supplementary Fig. 8). As shown in Fig. 7a, the statistical results of PSF measurements from CZ defect spots confirmed that the resolution of the *super*SIL system at room temperature (153 ± 15 nm) is indistinguishable from that measured under cryogenic conditions (Fig. 3a, c).

We compared the performance of the *super*SIL microscope with that of a state-of-the-art, off-the-shelf STORM microscope (ZEISS Elyra equipped with an Alpha Plan-Apochromat ×100/1.46 NA oil immersion DIC objective). To reach quasi-equilibrium between the fluorescent on and off states of the EGFP molecules, power densities of 0.349 and 1.1 kW cm^−2^ (*λ* = 488 nm) were delivered at the sample plane in the *super*SIL and off-the-shelf microscopes, respectively. The ~3-fold lower power density delivered to the sample in the *super*SIL microscope was required to avoid fast photobleaching. Both microscopes were equipped with the same EMCCD type (Andor, iXon+ DU-897). To compensate for lower power density and obtain similar image intensity as that of the off-the-shelf Elyra system, the linear EMCCD gain in the *super*SIL microscope was set 3-fold higher. In the *super*SIL set-up, we again used the 512/25 nm bandpass emission filter (57% transmission). In the Elyra system we used a 535/50 nm bandpass (64% transmission) (Supplementary Fig. 6A, C).

We imaged live *E. coli* cells expressing the antibacterial peptide ABC transporter McjD fused with EGFP^33,35–37^. Images of non-fluorescent *E. coli* cells expressing McjD transporter with no EGFP are shown in Supplementary Fig. 9. Typical wide-field images of EGFP-stained bacterial features from the *super*SIL and off-the-shelf microscopes are shown in Fig. 7b. Of note, the combination of a *super*SIL with a ×100 dry objective lens results in a magnification of ×471, nearly 5 times greater than a conventional ZEISS ×100 oil immersion objective.

Figure 7c shows representative STORM images and corresponding molecular localization precision histograms. The predicted localization improvement of the *super*SIL set-up with respect to the Elyra system predicted by (NA$\sqrt N$)/(NA′$\sqrt {N\prime }$) under the above set-ups is 1.6. In agreement within errors with this prediction, the results reveal a ~2-fold enhancement in localization precision using the *super*SIL (15.4 ± 4.1 nm) compared with the off-the-shelf system (31.3 ± 9.3 nm). In this case, the higher 2.17 NA of the *super*SIL contributes 1.5-fold and the increase in photon collection 1.13-fold, with 0.06-fold being lost by the 25 nm bandwidth of the bandpass filter.

Comparison of localization precision obtained with the *super*SIL system at room temperature (15.4 ± 4.1 nm) (Fig. 7c) with that obtained under cryogenic conditions (7.7 ± 3.2 nm) (Fig. 3f) shows that the higher photon budget under cryogenic conditions improves resolution by ~2-fold, consistent with our results when comparing the two off-the-shelf systems we used as controls. Indeed, both off-the-shelf systems deliver similar localization precisions at room (31.3 ± 9.3 nm) and cryogenic (35.7 ± 9.4 nm) (Supplementary Fig. 5D) temperatures because of the lower NA of the dry objective required for the latter. Allowing for differences in filter throughput and detector efficiency, the localization precision delivered by the 1.46 NA oil immersion lens with a collection efficiency of 36% with respect to the 0.9 NA dry lens with collection efficiency of 28% should have been 2-fold better, according to (NA$\sqrt N$)/(NA′$\sqrt {N\prime }$). Because we find approximately the same localization precision in both off-the-shelf systems, this shows that the enhanced photon budget afforded at cryogenic temperatures has compensated for the expected ~2-fold difference.

The field of view in the *super*SIL images is relatively small, (maximum size of 17.4 × 17.4 μm^2^ in one frame), the result of high magnification and finite camera chip size. The usable field of view can be extended to 60 × 60 μm^2^ by scanning the sample in the lateral plane (Supplementary Fig. 10). Imaging becomes impractical beyond this area due to residual imaging aberrations^17^. Furthermore, it can be more difficult to handle samples, and more aberrations are present when imaging away from the surface.

Sample structure and labelling density can affect resolution^48^. To ascertain the ultimate localization precision obtainable at room temperature with the off-the-shelf Elyra STORM system, we imaged standard DNA origami calibration samples (GattaQuant), which carried ATTO 647N dye molecules ~20 times brighter than EGFP^49^. As shown in Fig. 7d, results revealed a localization precision of 17.6 ± 8.1 nm, in line with the manufacturer’s specifications. Interestingly, the best localization precision we could extract from the specialist system using ATTO 647N was no better than the 15.4 ± 4.1 nm returned by the *super*SIL system using the much dimmer EGFP fluorophore, so we conclude that the *super*SIL microscope outperforms the off-the-shelf STORM system. Moreover, the *super*SIL microscope costs ~20 times less than the specialist STORM system. A comparison of *super*SIL microscopy versus fluid immersion microscopy is summarized in Table 1.

**Table 1:** Characteristics of *super*SIL microscopy versus fluid immersion microscopy

*Super*SIL microscopy	Fluid immersion microscopy
NAs in the range of 1.4–2.2 available	NA ~1.4
Super-high resolution	High resolution
×471 magnification	×100 magnification
Suitable for imaging under cryogenic conditions	Not suitable
Low cost	High cost

## Discussion

Our results show that by combining a *super*SIL and a low NA dry objective we have achieved our goal of improving 2-fold the resolution that can be achieved by STORM, using cell-friendly off-the-shelf equipment. Indeed, the localization precision and resolution attained are, to our knowledge, the best obtained to date on cell samples using a simple set-up. *Super*SIL-based super-resolution is not limited to STORM, but can be combined with other established super-resolution imaging techniques such as SIM^22^ and STED^20^. Importantly, the *super*SIL’s high NA eliminates the barrier to combine cryo-imaging with TIRF, paving the way to the application of vitrification to super-resolution imaging in mammalian cells.

During characterization of the *super*SIL set-up, we found an unexpected bonus. The ultra-high NA of *super*SIL and its enhanced photon collection properties also deliver a better resolution at room temperature than the off-the-shelf STORM system. This means that, even if cryogenic conditions are unneccesary, e.g. when imaging live cells, a simple add-on based around the use of *super*SILs would enable any non-expert with a basic fluorescence microscope to achieve state-of-the-art super-resolution at low cost. In conditions where long pass filters can be used in conventional set-ups, the bulk of the increase in resolution that can be obtained from *super*SILs is due to the larger NA. This is because the increased photon collection of the *super*SIL will be cancelled out by the losses in the 25 nm bandpass filters required to minimize chromatic aberration. The latter is less likely to be an issue in multi-colour imaging, where bandpass filters are typically required in any case. We used *super*SILs in an upright microscope, but a simple modification of the *super*SIL assembly would easily enable the use of *super*SILs in inverted microscopes. The method therefore can overcome the costs and complexity inherent in specialist super-resolution set-ups by circumventing the use of expensive objectives, intricate multi-stage illumination paths, specialized sample stages, and high power lasers.

Given these advantages, we propose that low-cost *super*SIL technology has the potential to greatly extend the scope and the reach of super-resolution microscopy in cell biology. Furthermore, it has the potential to revolutionize cryo-CLEM, the use of which has been limited by the resolution mismatch between EM and super-resolution, largely restricting the optical microscopy element of the method to general identification of regions of interest. By delivering close to 10 nm resolution under cryogenic conditions, cryo-*super*SIL imaging is poised to bridge the resolution gap between fluorescence microscopy and EM, finally allowing sample registration at the nanoscale. Possible schemes for CLEM include imaging of cryogenically-sectioned samples by cryo-*super*SIL STORM and transmission EM, or the use of focus ion beam scanning EM to image and produce lamellae suitable for the cryo-*super*SIL microscope. Given the possibility of scaling down *super*SILs to very small and custom-variable sizes, it is possible to envisage these devices being incorporated into EM systems, allowing true correlative microscopy without the need to move the sample and relocate areas of interest.

In summary, the very high NA and efficient photon collection properties of *super*SILs deliver an unprecedented resolution to cell biology, simply and inexpensively. *Super*SIL technology has the potential not only to transform super-resolution microscopy and CLEM, but also to increase the resolution attainable in any non-specialist laboratory at low-cost.

## Methods

### *Super*SIL microscope

The light source was a laser beam combiner (Omicron, LightHUB), including 488 and 642 nm laser lines, and a 470 nm collimated LED (Thorlabs, M470L3-C5). For the single colour imaging of McjD-EGFP and PH1735-EGFP *E. coli* cells, the filter sets consisted of a 484 nm beam splitter (Semrock, FF484-FDi0) as dichroic filter, and a 512/25 nm bandpass filter (Semrock FF01-512/25-25) as emission filter. For the two-colour imaging of McjD-EGFP and membrane- DiSC_3_(5) *E. coli* cells, the filter sets consisted of quad-edge dichroic beam splitter (Semrock, Di01-R405/488/543/635-25×36) as dichroic filter and quad-band bandpass filter (Semrock, FF01-446/523/600/677-25) as emission filter, also used in the DNA origami nanoruler measurement.

### *Super*SIL assembly

The 1 mm diameter *super*SILs (Knight Optical Ltd., UK) were made of cubic zirconia (ZrO_2_), the cubic crystalline form of zirconium dioxide (ZrO_2_). The lenses’ refractive index is 2.17 and the Abbe number is 33.54 at the wavelength of 512 nm. This provides a high refractive index with medium dispersion suitable for *super*SIL microscopy. The assemblies were characterized by use of a coordinate measuring machine (OGP SmartScope ZIP 250 Coordinate Measuring Machine) to ensure the angle between the platinum disk and the *super*SIL’s flat surface is <1° (Supplementary Fig. 2B).

### STORM data analysis

ZEISS ZEN software was used to process and render STORM images. In STORM image processing, various peak mask sizes were applied depending on pixel resolution and PSF size in each raw data set. The fit model was a two-dimensional Gaussian fit, and only single emitters from fluorophores were taken into account, whereas all multiple emitters were discarded. Following localization, displacements of molecules from drifts in the reconstructed images were corrected using feature detection and cross correlation. The counts from the raw images were first converted to the signal counts by deducting bias offset. Then the signal counts were converted to signal electrons by multiplying with the preamplifier gain. Finally, the signal electrons were converted to photon numbers by adding the detector electron-multiplying gain.

### Sample preparation

*Bacterial cell culture and staining*: *E. coli* strain C43 expressing McjD fused with EGFP were prepared for imaging. Briefly, the overnight starter culture was diluted 1:100 in fresh LB media supplemented with 50 μg ml^−1^ kanamycin and grown at 37 °C until an optical density at 600 nm (O.D._600_) of 0.6 was achieved. Then the expression of McjD was induced by adding IPTG (Isopropyl β-D-1-thiogalactopyranoside) to a final concentration of 1 mM at 25 °C overnight. The cells were spun down before freezing. The EGFP counts were measured using a Spectramax microplate reader (Molecular Devices) from 1 ml of culture re-suspended in 200 μl PBS (Thermofisher). For the analysis, the bacterial pellet was re-suspended in 2 ml of PBS to an optical density (O.D._600_) of ~0.6 and kept on ice until plunge-freezing. For two-colour imaging, *E. coli* expressing McjD-EGFP were re-suspended in 250 μl PBS to O.D._600_ ~ 0.6 and centrifuged at 10,000*g* for 5 min to wash out residues of culture medium. The pellet was then re-suspended in 25 μl of 100 nM DiSC_3_(5) in DPBS and incubated for 15 min on ice. 2.5 μl of sample were applied to the flat surface of each *super*SIL immediately prior to plunge-freezing.

*Mammalian cell culture and staining*: All reagents unless otherwise stated were from Thermo Scientific, UK. CHO cells expressing wtEGFR under an inducible Tet-ON promoter were grown in 5% CO_2_ in air at 37 °C in phenol-red free Dulbecco’s Modified Eagle medium (DMEM) supplemented with 10% (v/v) fetal bovine serum, 2 mM glutamine, 100 μg ml^−1^ hygromycin B and 100 μg ml^−1^ geneticin. All cells used were regularly tested for mycoplasma contamination. Cells were seeded at a density of 10^5^ ml^−1^ on superSILS passivated with PEG-BSA nanogel as described previously^50,51^. Briefly, superSILs were etched with Piranha solution for 10 min, and thoroughly rinsed. Priming was performed for 5 min with Vectabond reagent (Vectorlabs) diluted 1:50 in acetone, followed by thorough rinsing. PEG-BSA nanogel was applied for 1 h at 37 °C, rinsed twice with PBS, capped with 20 mg ml^−1^ BSA in PBS for 1 h 37 °C, quenched with 1 M Tris pH 8.0 for 15 min, followed by three washes in PBS. Cells were cultured for 48 h, rinsed, subjected to nutrient starvation for 2 h at 37 °C to wash out EGFR ligands from the serum and then labelled with 5 nM EGF conjugated to Alexa Fluor 488 (Thermo Scientific) for 30 min at 4 °C. Clustering of EGFR was induced by a 5 min incubation at 37 °C prior to plunge-freezing.

*DNA origami nanorulers*: Samples were prepared according to manufacturer’s instructions. Briefly, superSILs were glow discharged for 120 s (negative) using a Quorum Q150T ES system, then washed 3 times with PBS, then immersed for 5 min in a solution of BSA-biotin 1 mg ml^−1^ in PBS and washed a further 3 times with PBS. Coating with neutravidin 1 mg ml^−1^ in PBS was also performed by immersion for 5 min, followed by three washes in PBS + 10 mM MgCl_2_ (immobilization buffer). 2.5 μl of DNA Origami diluted in immobilization buffer to a final concentration of 1:100 were applied to the flat surface of the *super*SILs, prior to blotting and plunge-freezing.

*Plunge freezing*: The superSILs were etched for 15 min with Piranha solution (3:1 concentrated sulphuric acid, 30% H_2_O_2_, both from Sigma-Aldrich) then rinsed with plenty of water and left to air dry for 1 h. SuperSILs were then glow-discharged for 120 s (negative) prior to sample loading and freezing using a Quorum Q150T ES system. Samples were frozen by plunge-freezing using FEI Vitrobot MKIV according to manufacturer’s instructions. The chamber was equilibrated to 4 °C, 95% relative humidity. Blotting was performed manually.

### Reporting summary

Further information on experimental design is available in the Nature Research Reporting Summary linked to this article.

### Code availability

ZEN software was used to process and render STORM images in this work. The software is commercially available from Carl Zeiss Ltd.

## Supplementary information

Supplementary Information

Reporting Summary

## Data Availability

All relevant data are available from the authors upon request.
