# Supplementary material for: Solid immersion microscopy images cells under cryogenic conditions with 12 nm resolution
Source: Commun Biol. 2019 Feb 21;2:74. doi: 10.1038/s42003-019-0317-6 (PMC6385270; doi:10.1038/s42003-019-0317-6)
Supplement: Supplementary file 1 — Supplementary Information [file 42003_2019_317_MOESM1_ESM.pdf]

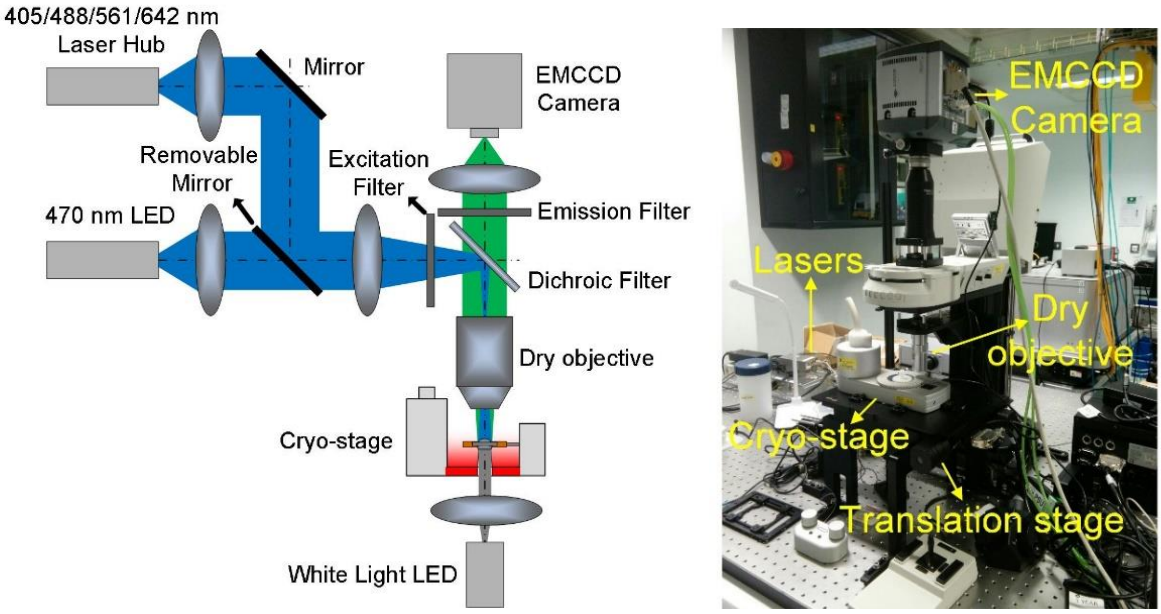

Supplementary Figure 1| The set-up of the *super*SIL microscope. Schematic (left) and a photo (right) of bench-top *super*SIL microscope.

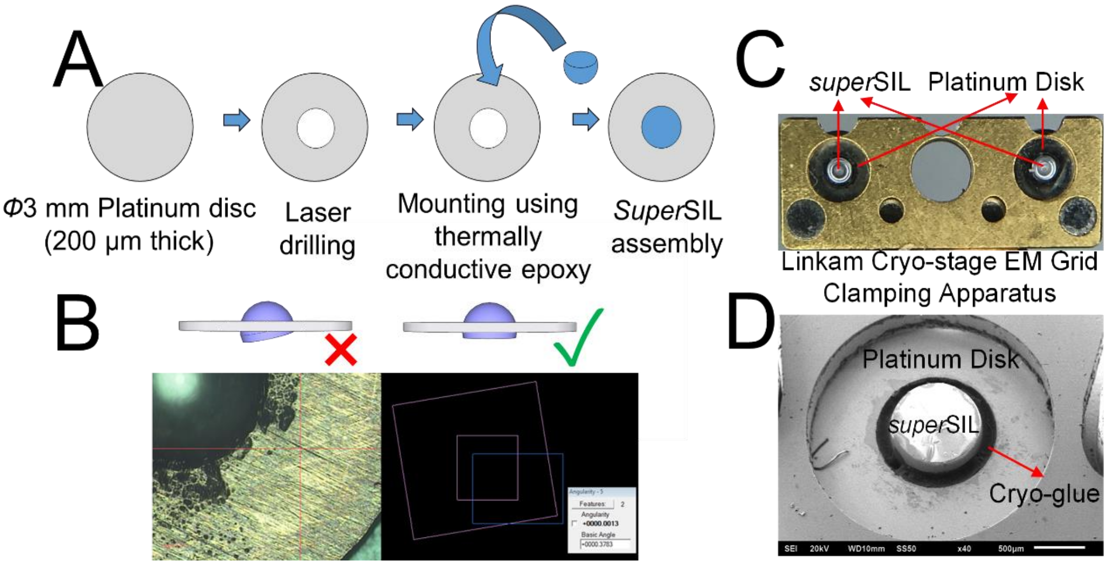

Supplementary Figure 2| Mechanical details of the *super*SIL assembly procedure. (A) Schematic of fabrication procedure of *super*SIL assembly; (B) Schematic of incorrect and correct positioning of a *super*SIL in an assembly, which was inspected using Coordinate Measuring Machine (CMM) characterization to ensure the angle between the Platinum disk and *super*SIL flat surface is < 1°. (C) Photo of two *super*SIL assemblies clamped in a standard EM grid clamping apparatus in Linkam CMS-196 cryo-stage. The flat surfaces of the *super*SILs are shown. (D) Scanning Electron Microscopy (SEM) image of one *super*SIL assembly, in which the epoxy (cryo-glue) linking the *super*SIL and the platinum disk is shown.

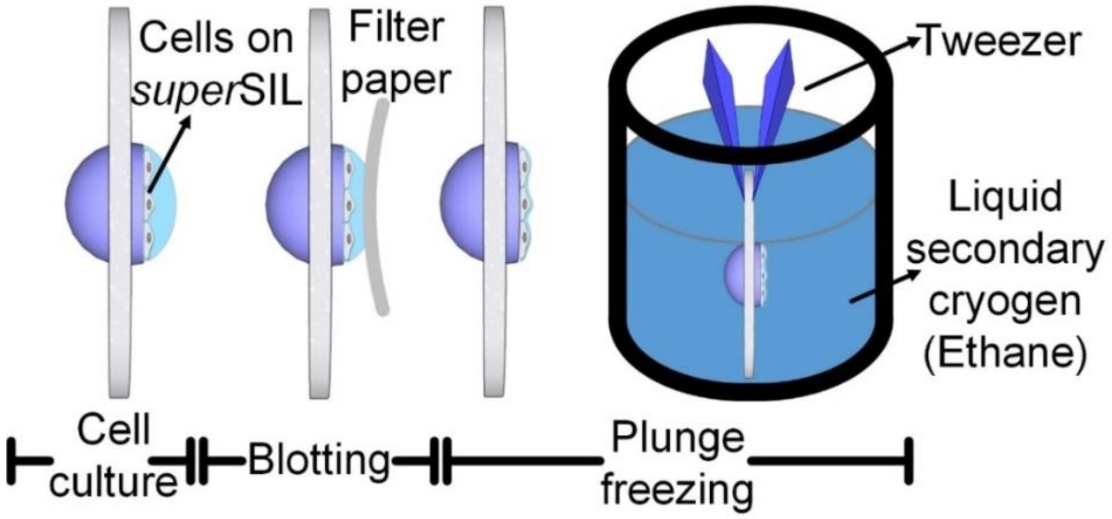

Supplementary Figure 3| Plunge freezing protocol. Schematic of plunge freezing procedure of mammalian cells on *super*SIL assembly. Bacterial cells were drop casted on the *super*SIL surface immediately prior to blotting

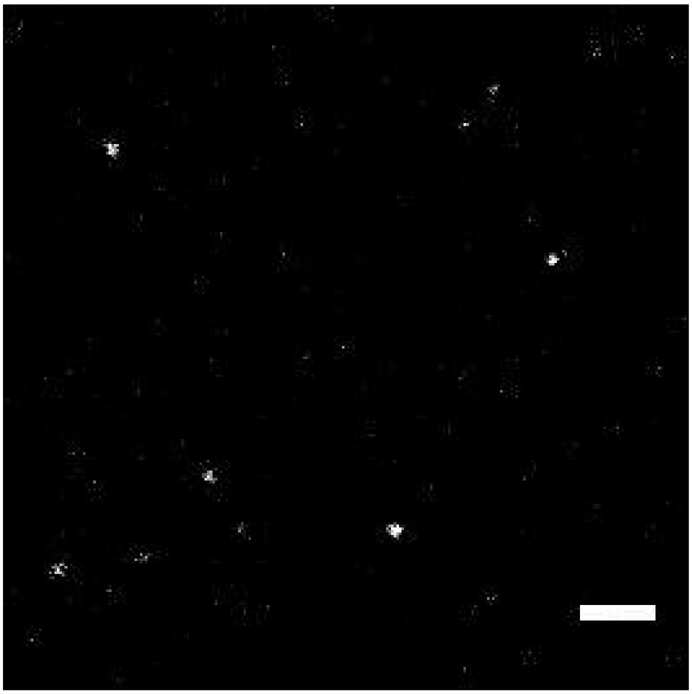

Supplementary Figure 4| *Super*SIL images of cubic zirconia defects. Representative wide-field *super*SIL FM images of CZ defects. Scale bar: 1 µm.

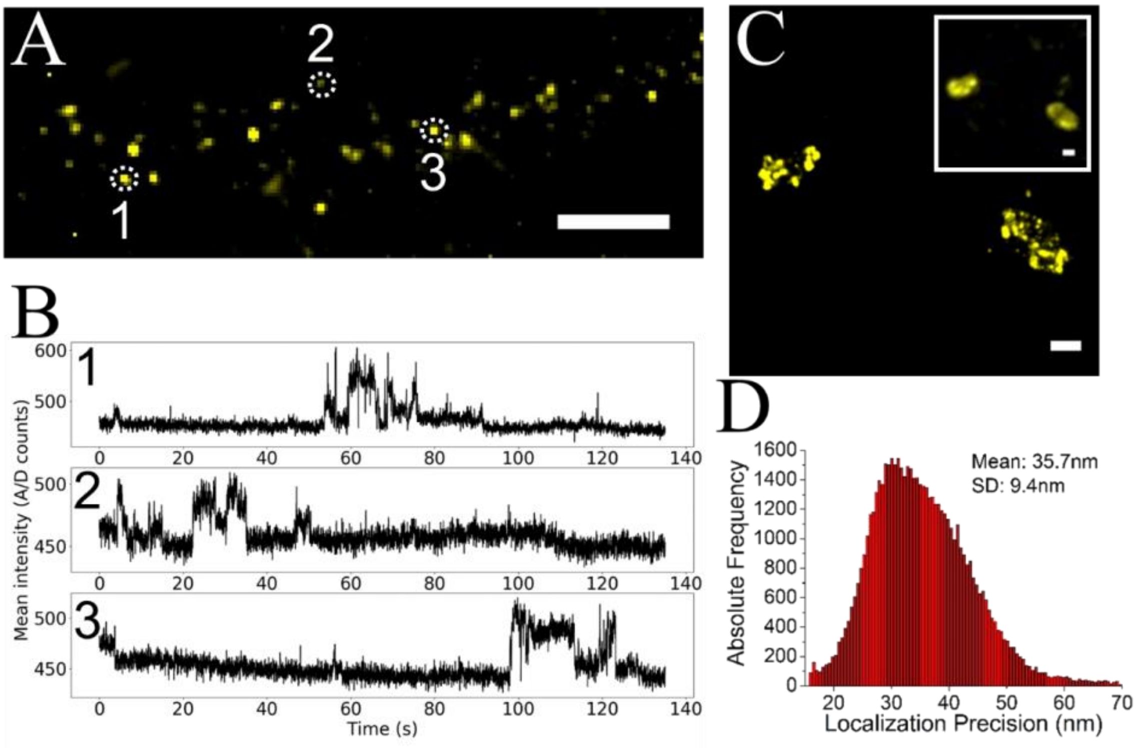

Supplementary Figure 5| Characteristic blinking from single EGFP molecules at cryogenic temperature. EGFP molecule blinking characterization at cryogenic temperature. (A) Representative single frame image of single molecules of EGFP in a time-lapse stack acquired in an FEI CorrSight microscope using a 0.9NA 40× dry objective at cryogenic temperature. EGFP molecules were drop casted on to a conventional copper EM grid and plunge frozen at cryogenic temperature. Scale bar: 5µm. (B) The respective intensity time traces exhibiting characteristic blinking from 3 locations indicated by the dotted circles in (A). (C) STORM image of frozen McjD-EGFP in *E.coli* cells imaged on the FEI CorrSight microscope at cryogenic termperature. The inset shows the wide-field fluorescence image of the cells. Scale bar: 1 µm. (D) The histogram of localization precision from (C) demonstrates (35.7 ± 9.4) nm localization precision.

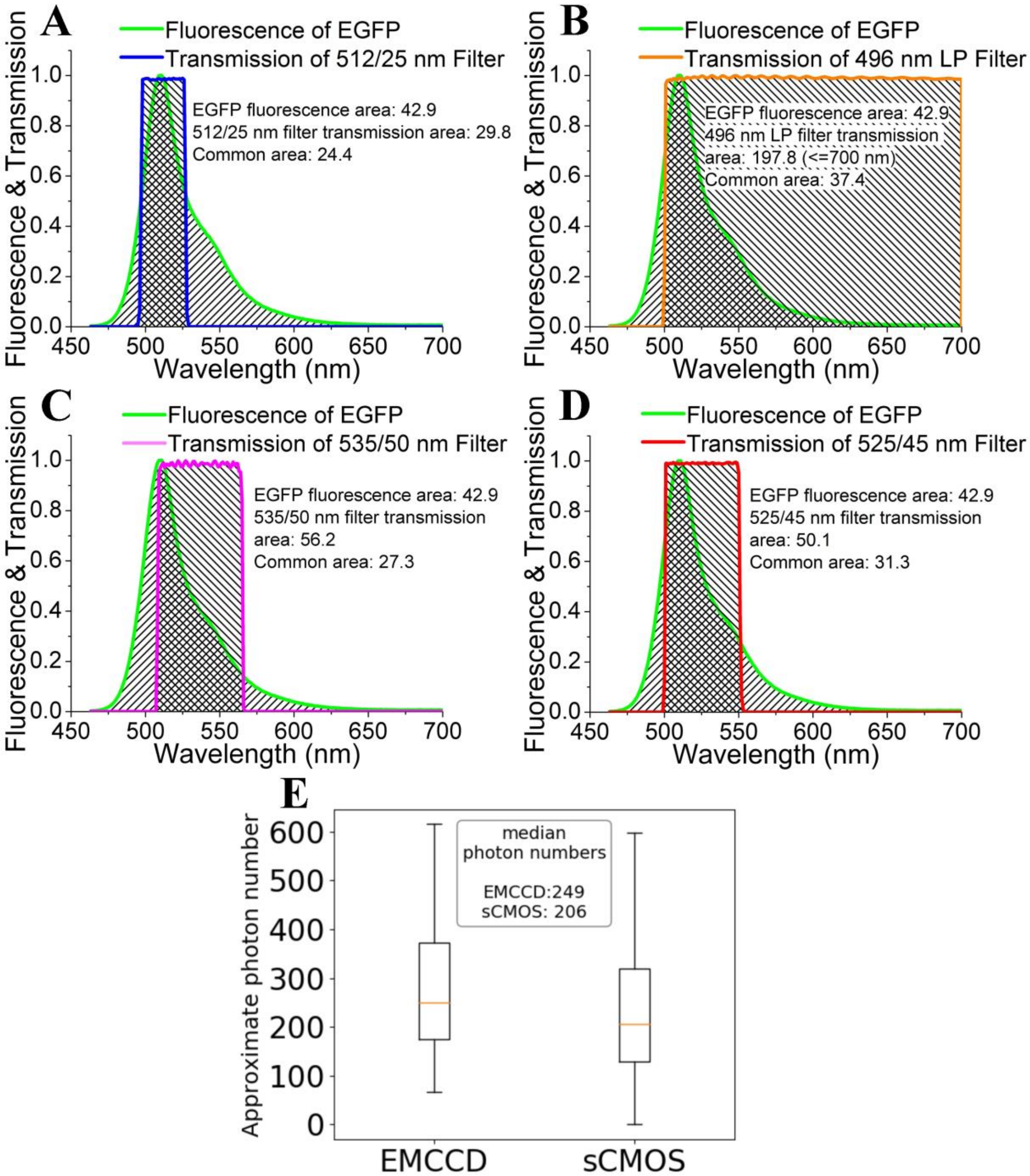

Supplementary Figure 6| Comparison of photon collection using different widths of bandpass emission filters. The deployment of narrow passband emission filters in fluorescence microscopy may cause the reduction of fluorescence emission. We characterized the collection capacity of EGFP emission in the *super*SIL microscope where a 512/25 nm bandpass filter (Semrock FF01-512/25-25) was used, a commercial microscope (FEI CorrSight) where a 496 nm long pass filter (Semrock FF01-496/LP-25) was used, and a commercial microscope (ZEISS, Elyra P.S1) where a 535/50 nm bandpass filter (ZEISS EF BP 535/50) was used. We also used a

525/45 nm bandpass filter (Semrock FF01-525/45-25), optimal for EGFP fluorescence collection. It is concluded that 57%, 87%, 64%, and 73% of EGFP emission could be collected in these four cases, demonstrating that careful consideration of the filter choices can minimize the photon budget reduction when a narrow bandpass filter is deployed. (A) Fluorescence of EGFP and the bandpass of a 512/25 nm filter used in *super*SIL microscope. 57% of the EGFP emission can be collected. (B) Fluorescence of EGFP and the bandpass of a 496 nm long pass filter used in the FEI CorrSight. 87% of the EGFP emission can be collected. (C) Fluorescence of EGFP and the bandpass of a 535/50 nm filter used in Elyra microscope. 64% of EGFP emission can be collected. (D) Fluorescence of EGFP and the bandpass of a 525/45 nm filter. 73% of EGFP emission can be collected. (E) Number of photons detected by the EMCCD used in the *super*SIL setup (Andor, iXon+ DU-897) and the sCMOS (Hamamatsu, ORCA-Flash4.0 V2) used in the FEI CorrSight from the same field of 100 nm fluorescent beads placed at the sample position of an upright microscope using the same objective lens (0.55 NA), same laser power, and same filters. ADU counts were collected in both detectors sequentially and subsequently converted to photons using the spec sheets for each detector. The plot shows the 25th and 75th quartiles with whiskers at 1.5x inter-quartile range, with the median values shown in orange.

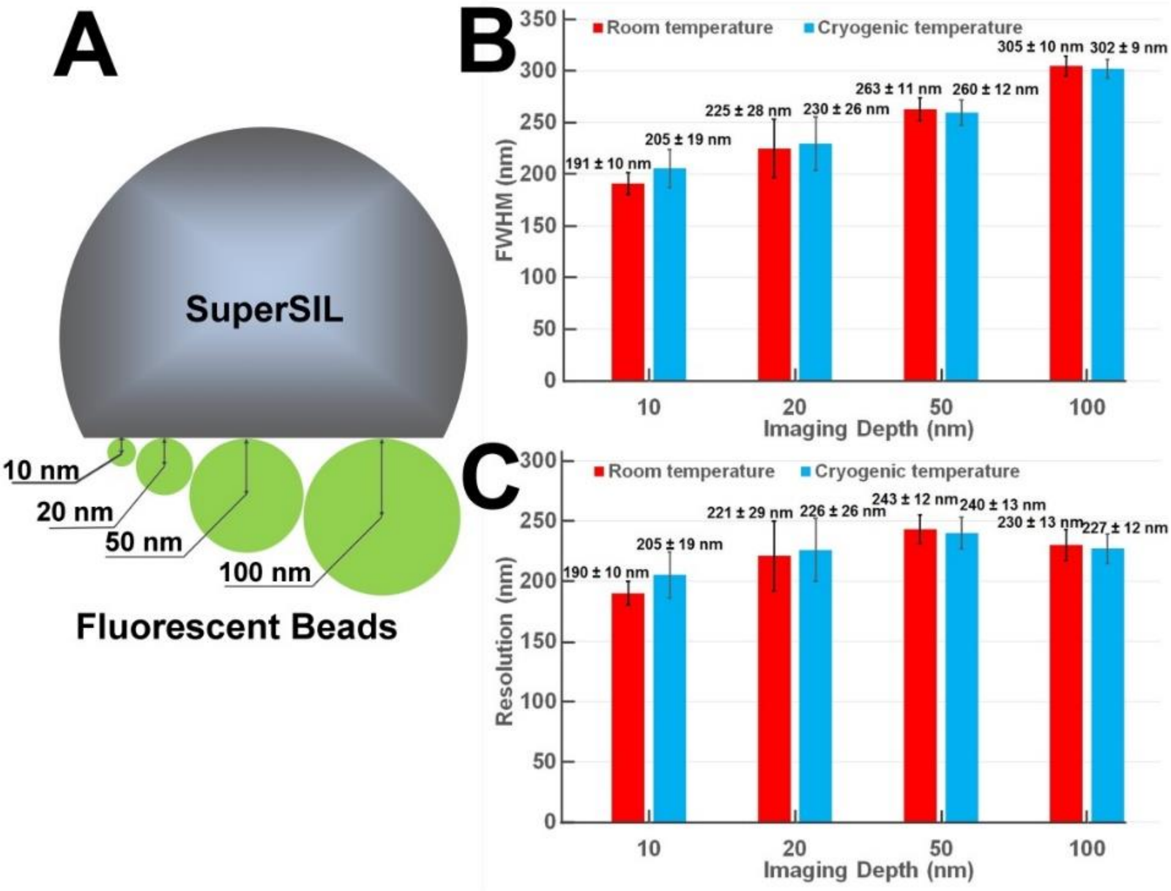

Supplementary Figure 7| Dependence of the resolution of the *super*SIL with depth. (A) Method cartoon: Fluorescent beads in the diameters of 20 nm, 40 nm, 100 nm, 200 nm were used to measure the FWHMs of bead images, corresponding to equatorial imaging depths of 10 nm, 20 nm, 50 nm and 100 nm. (B) FWHM and (C) resolution at the corresponding imaging depths after deconvolution. As expected, deconvolution does not affect the results from sub-diffraction-limited beads. Data are presented as mean ± standard deviation.

To investigate the depth of focus within which the resolution of the *super*SIL is not significantly deteriorated we compared images of 20, 40, 100 and 200 nm diameter fluorescent beads (ThermoFisher, TetraSpeck). The equatorial planes of these beads are separated by 10, 20, 50, and 100 nm from the aplanatic plane of the lens, thus these beads can be used as depth resolution rulers. The experimental set-up is described in the cartoon (**A**). We first measured the size of the PSF for each bead size (**B**). For the 20 nm and 40 nm beads, which are sub-diffraction limited, we observed a 25% increase in the FWHM of the measured PSF with respect to that found from single point emitters located on the flat surface the *super*SIL. We attribute this effect to the separation between the equatorial planes of the beads and the aplanatic plane of the *super*SIL [1]. As expected, the 100 nm and 200 nm beads, which are comparable to, or larger than, respectively, the theoretical 120 nm resolution of the *super*SIL system [1], returned

larger PSFs. The corresponding resolution was determined by correcting for the actual size of the beads in the PSF measurement. The image is a convolution of the PSF with the beads. Approximating them as Gaussians should be reasonable for our purposes [2]. The convolution gives:

$$FWHM_{meas}^2 = FWHM_{PSF}^2 + D_{bead}^2.$$

Taking D_{bead} as the bead diameter, we can calculate the PSF size, $FWHM_{PSF}$, from the measured size $FWHM_{meas}$. The results, shown in **(C)**, demonstrate that the resolution performance is degraded by an approximately constant value (± 16%) throughout the depth range of 10-100 nm. This value is smaller than the ± 38% experimental error in the localization precision (σ) found for molecules directly on the aplanatic surface of the *super*SIL. We thus conclude that, for up to 100 nm separation from the aplanatic plane, the resolution deterioration with depth is negligible.

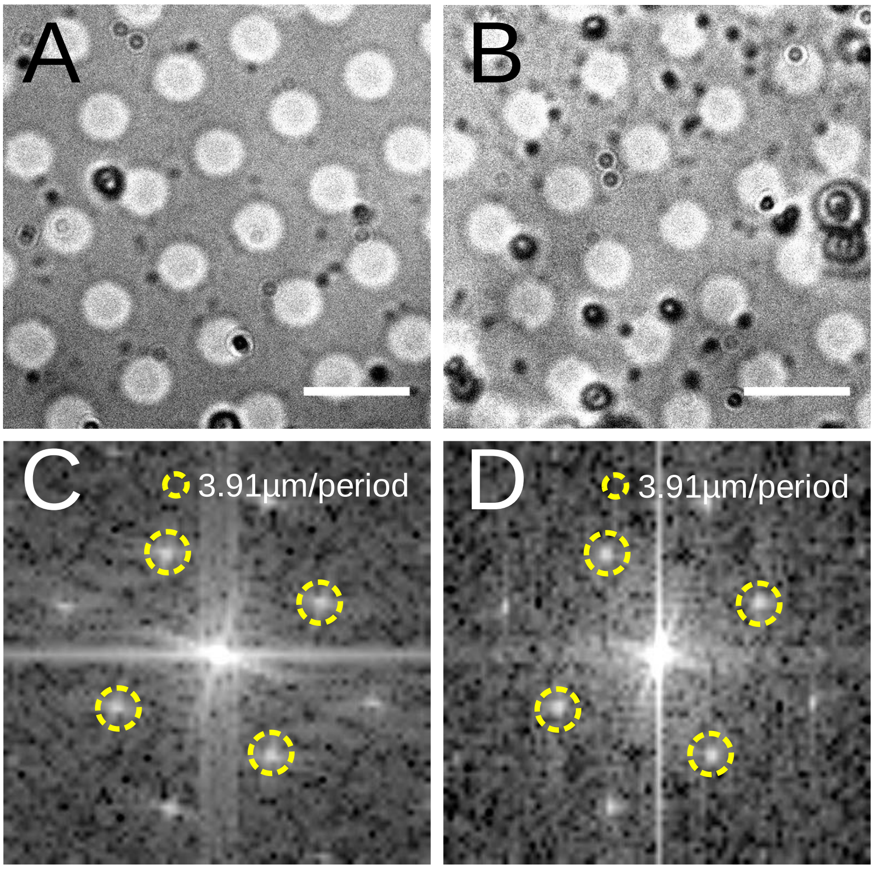

Supplementary Figure 8| *Super*SIL resolution as a function of temperature. Transmission bright-field image of a Quantifoil 200 mesh 2/2 holey carbon grid and their Fourier transforms at (**A, C**) room temperature and (**B, D**) cryogenic temperature. Scale bar: 5 µm. The Fourier transforms show that the holey carbon structures have the same period, i.e. 3.91 µm, at room temperature (C) and cryogenic temperature (D), indicating the changes of the optical parameters of the *super*SILs at cryogenic temperature are negligible, compared to those at room temperature.

At cryogenic temperature, the spectral dispersion and thermal expansion properties of cubic zirconia (CZ) may change compared with those at room temperature, and this may alter the optical parameters, such as the magnification/focal length, of CZ *super*SILs. We examined *super*SIL magnification at both room and cryogenic temperature using standard EM grids (Quantifoil AGS173-1) as test samples. The bright-field images of the same EM grid taken from the *super*SIL FM system at room and cryogenic temperatures show the same measured period from the holey structures on carbon film, therefore we concluded the spectral dispersion and thermal expansion changes at cryogenic temperature were negligible. The calibration of *super*SIL magnification allowed us to evaluate precisely the resolution, and more importantly, it indicates that the *super*SIL FM system should give similar resolution at room and cryogenic temperature.

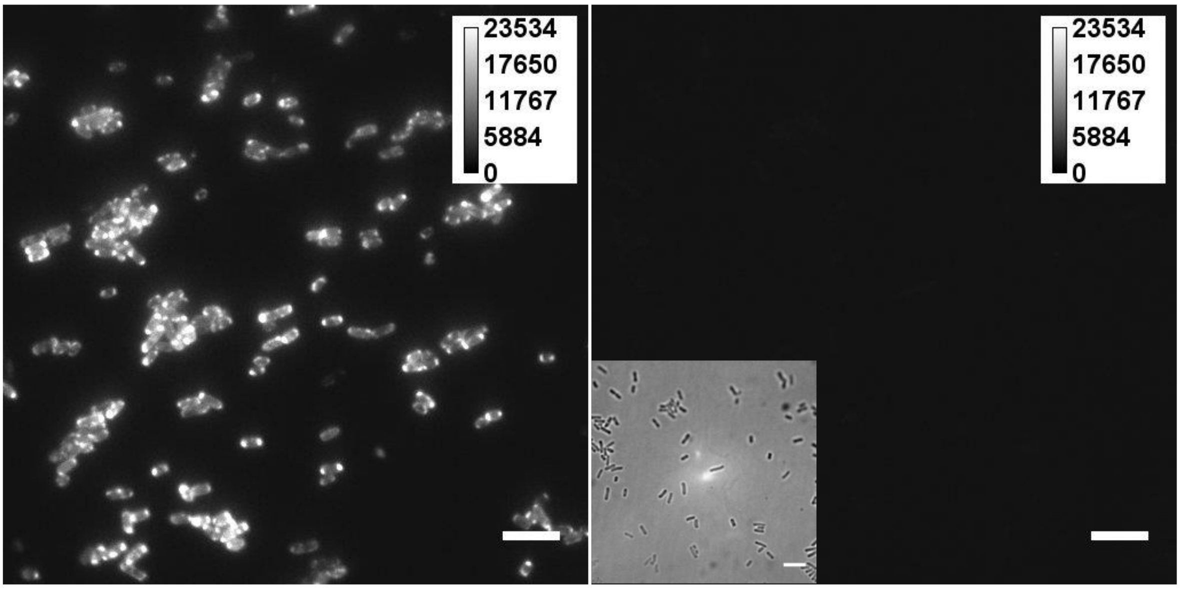

Supplementary Figure 9| Control measurement of EGFP fluorescence. EGFP fluorescence image and negative control measurement. (*Left*) McjD-EGFP *in E.coli* cells and (*right*) the negative control cells without EGFP fusion were imaged at room temperature. A transmission bright-field image of the cells for negative control is shown in the small inset. The auto-fluorescence from the cells is negligible compared to that of EGFP, demonstrating EGFP is the fluorophore responsible for the blinks in super-resolution imaging. Imaging was implemented in a ZEISS Elyra microscope using 100× 1.46 NA objective lens in the same imaging condition.

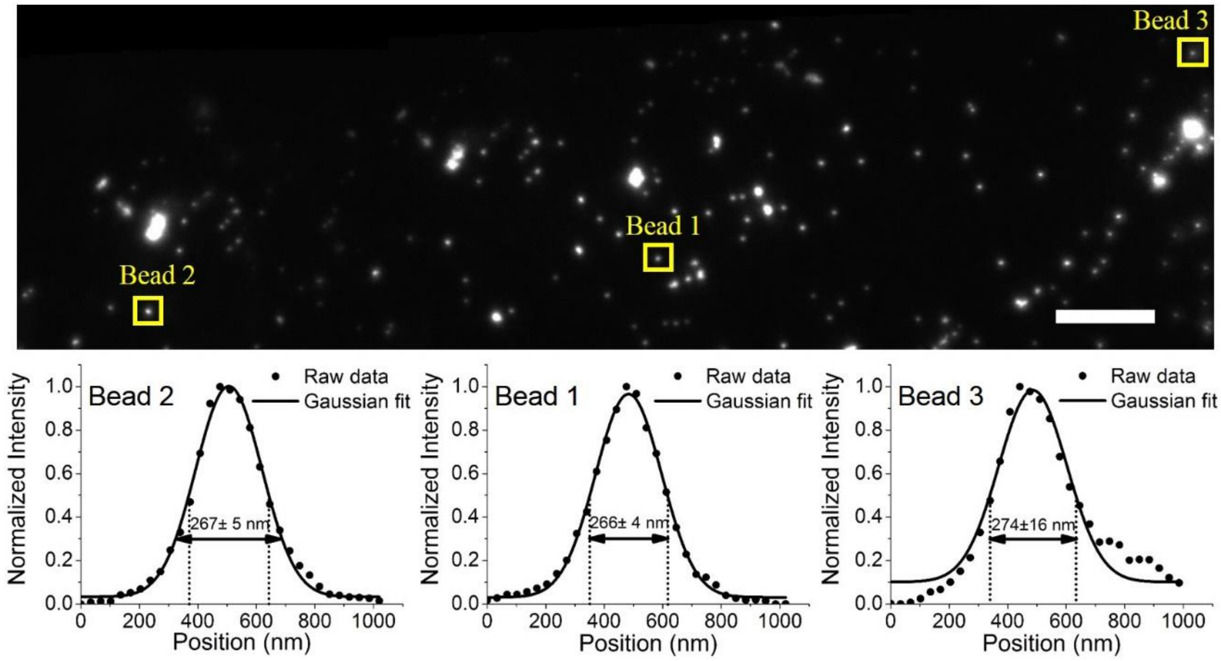

Supplementary Figure 10| Measurement of full usable field of view. Full usable field of view in the *super*SIL microscope. (*top*) Composite image of 60.42×17.41 µm (1777×512 pixels) obtained from stitching a series of images captured by tile scanning the sample in one direction. The sample was a field of 100 nm fluorescent beads. Scale bar: 5µm. (*bottom*) Profiles and corresponding FWHM sizes from the three fluorescence beads indicated by the yellow boxes in the image above. Two fluorescent beads, i.e. beads 2 and 3, at the peripheral of the image show similar FWHM sizes to that of bead 1 located in the centre of the image, indicating aberrations are insignificant at the periphery of the whole field of view.

Supplementary Table 1. ***Super*****SIL materials options and their predicted effective depth of foci**

Material	Refractive index at 512 nm	Minimum *NA* of matching dry objective	Effective *NA*	Resolution (nm)	Effective super-resolution imaging depth (nm)
Cubic Zirconia	2.17	0.46	2.16	118	100
S-LAH79 (glass)	2	0.5	2	128	118
Sapphire	1.77	0.56	1.77	145	150
BK7 (glass)	1.52	0.66	1.52	168	204
Quartz	1.46	0.68	1.46	175	221

Supplementary References

1. Kim, W.-C., et al., *Investigation on achieving super-resolution by solid immersion lens based STED microscopy.* Optics Express, 2017. **25**(14): p. 16629-16642.

2. Wu, Q., et al., *Realization of numerical aperture 2.0 using a gallium phosphide solid mmersion lens.* Applied Physics Letters, 1999. **75**(26): p. 4064-4066
