# Supplementary material for: Solid immersion microscopy images cells under cryogenic conditions with 12 nm resolution
Source: Commun Biol. 2019 Feb 21;2:74. doi: 10.1038/s42003-019-0317-6 (PMC6385270; doi:10.1038/s42003-019-0317-6)
Supplement: Supplementary file 2 — Reporting Summary [file 42003_2019_317_MOESM2_ESM.pdf]

nature research

Corresponding author(s): Marisa Martin-Fernandez
Last updated by author(s): Jan 11, 2019

Reporting Summary

Nature Research wishes to improve the reproducibility of the work that we publish. This form provides structure for consistency and transparency in reporting. For further information on Nature Research policies, see Authors & Referees and the Editorial Policy Checklist.

Statistics

For all statistical analyses, confirm that the following items are present in the figure legend, table legend, main text, or Methods section.

n/a	Confirmed	
☐	☒	The exact sample size (*n*) for each experimental group/condition, given as a discrete number and unit of measurement
☐	☒	A statement on whether measurements were taken from distinct samples or whether the same sample was measured repeatedly
☐	☒	The statistical test(s) used AND whether they are one- or two-sided<br>*Only common tests should be described solely by name; describe more complex techniques in the Methods section.*
☒	☐	A description of all covariates tested
☒	☐	A description of any assumptions or corrections, such as tests of normality and adjustment for multiple comparisons
☐	☒	A full description of the statistical parameters including central tendency (e.g. means) or other basic estimates (e.g. regression coefficient) AND variation (e.g. standard deviation) or associated estimates of uncertainty (e.g. confidence intervals)
☒	☐	For null hypothesis testing, the test statistic (e.g. *F*, *t*, *r*) with confidence intervals, effect sizes, degrees of freedom and *P* value noted<br>*Give P values as exact values whenever suitable.*
☒	☐	For Bayesian analysis, information on the choice of priors and Markov chain Monte Carlo settings
☒	☐	For hierarchical and complex designs, identification of the appropriate level for tests and full reporting of outcomes
☒	☐	Estimates of effect sizes (e.g. Cohen's *d*, Pearson's *r*), indicating how they were calculated

Our web collection on statistics for biologists contains articles on many of the points above.

Software and code

Policy information about availability of computer code

Data collection	Image data from bench-top superSIL microscope was collected using open source software Micro-Manager. Image data from ZEISS Elyra microscope was collected using commercially available software ZEN from Carl Zeiss. Image data from FEI CorrSight microscope was collected using commercially available software MAPS from FEI. All relevant data are available from the authors upon reasonable request.
Data analysis	ZEN software was used to process and render STORM images in this work. The software is commercially available from Carl Zeiss Ltd. All relevant data are available from the authors upon reasonable request.

For manuscripts utilizing custom algorithms or software that are central to the research but not yet described in published literature, software must be made available to editors/reviewers. We strongly encourage code deposition in a community repository (e.g. GitHub). See the Nature Research guidelines for submitting code & software for further information.

Data

Policy information about availability of data

All manuscripts must include a data availability statement. This statement should provide the following information, where applicable:

- Accession codes, unique identifiers, or web links for publicly available datasets
- A list of figures that have associated raw data
- A description of any restrictions on data availability

All relevant data are available from the authors upon reasonable request.

Field-specific reporting

Please select the one below that is the best fit for your research. If you are not sure, read the appropriate sections before making your selection.

☒ Life sciences ☐ Behavioural & social sciences ☐ Ecological, evolutionary & environmental sciences

For a reference copy of the document with all sections, see nature.com/documents/nr-reporting-summary-flat.pdf

Life sciences study design

All studies must disclose on these points even when the disclosure is negative.

Sample size	Bilogiocal samples were not used for statistical purposes or to draw conclusions about the features of the biological system that was being imaged but just to illustrate the potentiality of the superSIL technique, therefore we didn't take into account statistical considerations about sample size and no statistical analysis were performed on the samples, beyond reporting the localisation precision of detected single-molecule features or assessing measurement resolution with standard methods. The statistics of the latter were reported in the body of the paper.
Data exclusions	As no statistical analysis was performed about biological features of the samples, no data were excluded
Replication	As no statistical analysis was performed about biological features of the samples, no validation experiments were performed. The validation of the performance of the microscope using non-biological samples is described at length in the body of the paper
Randomization	Not applicable due to the lack of randomizable treatments/interventions in the experimental plan.
Blinding	Not applicable due to the lack of randomizable treatments/interventions in the experimental plan.

Reporting for specific materials, systems and methods

We require information from authors about some types of materials, experimental systems and methods used in many studies. Here, indicate whether each material, system or method listed is relevant to your study. If you are not sure if a list item applies to your research, read the appropriate section before selecting a response.

Materials & experimental systems

n/a	Involved in the study	
☒	☐	Antibodies
☐	☒	Eukaryotic cell lines
☒	☐	Palaeontology
☒	☐	Animals and other organisms
☒	☐	Human research participants
☒	☐	Clinical data

Methods

n/a	Involved in the study	
☒	☐	ChIP-seq
☒	☐	Flow cytometry
☒	☐	MRI-based neuroimaging

Eukaryotic cell lines

Policy information about cell lines

Cell line source(s)	Chinese Hamster Ovary (CHO) cells stably transfected with EGFR variants under a Tet-ON promoter (described in Macdonald, J. L. & Pike, L. J. Heterogeneity in EGF-binding affinities arises from negative cooperativity in an aggregating system. Proc Natl Acad Sci U S A 105, 112-117, doi:10.1073/pnas.0707080105 (2008) and Macdonald-Obermann, J. L. & Pike, L. J. The intracellular juxtamembrane domain of the epidermal growth factor (EGF) receptor is responsible for the allosteric regulation of EGF binding. J Biol Chem 284, 13570-13576, doi:10.1074/jbc.M109.001487 (2009)) were a gift from Prof Linda Pike, Washington University, St. Louis.
Authentication	Cell lines were not authenticated
Mycoplasma contamination	Cells were tested and confirmed negative for mycoplasma using standard DAPI stain and/or Lookout Mycoplasm PCR Detection Kit (Sigma, Cat no. MP0035)
Commonly misidentified lines (See ICLAC register)	CHO cell lines are not listed in the misidentified cell registry
